# Life-Threatening Eosinophilic Granulomatosis With Polyangiitis During Omalizumab Therapy for Chronic Urticaria: Missed Diagnosis, Induction, or Unmasking?

**DOI:** 10.7759/cureus.115576

**Published:** 2026-09-01

**Authors:** Panagiotis Zymaris, Athina Georgopoulou, Aikaterini Tzioutzia, Athanasios Zacharias, George Margaritopoulos, Evdokia Sourla, Afroditi Boutou, Georgia Pitsiou

**Affiliations:** 1 Department of Respiratory Failure, General Hospital of Thessaloniki “G. Papanikolaou”, Aristotle University of Thessaloniki, Thessaloniki, GRC; 2 Department of Respiratory Medicine, General Hospital of Thessaloniki “G. Papanikolaou”, Aristotle University of Thessaloniki, Thessaloniki, GRC

**Keywords:** chronic urticaria, diffuse alveolar hemorrhage, eosinophilia, eosinophilic granulomatosis with polyangiitis, omalizumab, vasculitis

## Abstract

Eosinophilic granulomatosis with polyangiitis (EGPA) is a rare form of vasculitis characterized by late-onset asthma, eosinophilia, and systemic manifestations involving small- and medium-sized vessels. We report a severe case of EGPA occurring during treatment with omalizumab in a patient diagnosed with chronic urticaria.

A 67-year-old woman presented with acute dyspnea, epistaxis, hemoptysis, and fatigue. Her medical history included late-onset asthma and chronic urticaria with persistent eosinophilia and elevated IgE levels, under treatment with omalizumab. Laboratory investigation revealed eosinophilia and elevated inflammatory markers, while chest computed tomography demonstrated bilateral ground-glass opacities. Bronchoalveolar lavage showed marked eosinophilia and progressively hemorrhagic sequential aliquots, confirming diffuse alveolar hemorrhage. Autoimmune panel was positive for MPO-p-ANCA, supporting the diagnosis of EGPA. The patient was initially treated with high-dose intravenous methylprednisolone, followed by rituximab. Despite treatment initiation, the patient rapidly deteriorated, requiring endotracheal intubation and admission to the intensive care unit. Treatment was escalated with therapeutic plasma exchange and intravenous cyclophosphamide. After a prolonged hospitalization, the patient gradually recovered and was transferred for rehabilitation.

This case report highlights the diagnostic challenges of EGPA and the importance of early recognition. Whether omalizumab unmasked, coincided with, or induced EGPA remains uncertain. Clinicians should investigate unexplained, persistent eosinophilia as it could be an expression of a systemic disease.

## Introduction

Eosinophilic granulomatosis with polyangiitis (EGPA), originally described by J. Churg and L. Strauss in 1951, is a rare systemic vasculitis characterized by small- and medium-vessel involvement, eosinophilia, and late-onset asthma, affecting fewer than 15 adults per million [1]. EGPA lies at the intersection of hypereosinophilic disorders and anti-neutrophil cytoplasmic antibody (ANCA)-associated diseases, and the clinical findings reflect both eosinophil-mediated tissue infiltration and necrotizing vessel inflammation [2]. Due to the systemic nature and the overlapping stages of the disease, the clinical manifestation can be variable, making the diagnosis particularly challenging [3]. Omalizumab is a recombinant humanized monoclonal antibody against immunoglobulin E (IgE), indicated for IgE-mediated severe asthma, but has also been proven useful for many conditions accompanied by high IgE levels, like chronic urticaria and atopic dermatitis [4-6].

In this report, we present the case of a patient developing EGPA while receiving treatment with omalizumab for chronic urticaria, where rapid progression led to a fulminant, life-threatening clinical course requiring endotracheal intubation and intensive care unit admission.

## Case presentation

A 67-year-old Caucasian woman presented to the Emergency Department complaining of recent-onset dyspnea. She also reported bouts of epistaxis, small-volume hemoptysis, and fatigue over the preceding month. Auscultation revealed bilateral crackles, oxygen saturation was 84% on room air, and axillary temperature was 37.5°C.

Her medical history included atrial fibrillation, dyslipidemia, arterial hypertension, chronic sinusitis, anxiety disorder, and late-onset asthma treated with budesonide/formoterol. She also reported chronic urticaria with a persistently elevated eosinophil count (>1.5 K/μL) and serum IgE levels (>300 IU/mL), for which she was treated with omalizumab (300 mg every four weeks) for the last one year, initiated by a dermatologist. In addition, she had undergone a thyroidectomy 15 years earlier for Hashimoto’s thyroiditis. Her regular medication also included rivaroxaban, flecainide, ezetimibe, irbesartan, bilastine, azelastine/fluticasone nasal spray, levothyroxine, and sertraline. The patient had been vaccinated within the previous year against respiratory syncytial virus, herpes zoster virus, and influenza.

Upon admission, a comprehensive laboratory evaluation was performed, including a complete blood count, renal and hepatic biochemical markers, inflammatory markers, an autoimmune screening panel, urinalysis, molecular testing for clonal hematologic disorders, and stool samples for parasitology screening. Baseline laboratory results showed eosinophilia (2.47 K/μL, 17%) and high C-reactive protein (21.9 mg/dL, normal values <0.5 mg/dL). Hematological evaluation, including JAK2 V617F and BCR::ABL1 testing, conventional karyotyping, and fluorescence in situ hybridization (FISH) for PDGFRA, PDGFRB, and FGFR1 rearrangements, was negative. Moreover, next-generation sequencing detected no pathogenic variants or chimeric transcripts. Stool studies for parasitic infection were negative.

The chest X-ray followed by a chest computed tomography revealed bilateral ground-glass opacities with areas of consolidation, consistent with diffuse alveolar hemorrhage (Figure 1). The patient was also subjected to fiberoptic bronchoscopy with bronchoalveolar lavage (BAL). BAL differential cell count showed reduced macrophages (12%), marked neutrophilia (66%), and eosinophilia (17.3%). Sequential BAL aliquots were progressively more hemorrhagic, and no specific source of bleeding was identified. Although hemorrhage was confirmed, no hemosiderin-laden macrophages were identified, likely due to the low macrophage count in the sample. 

**Figure 1:**
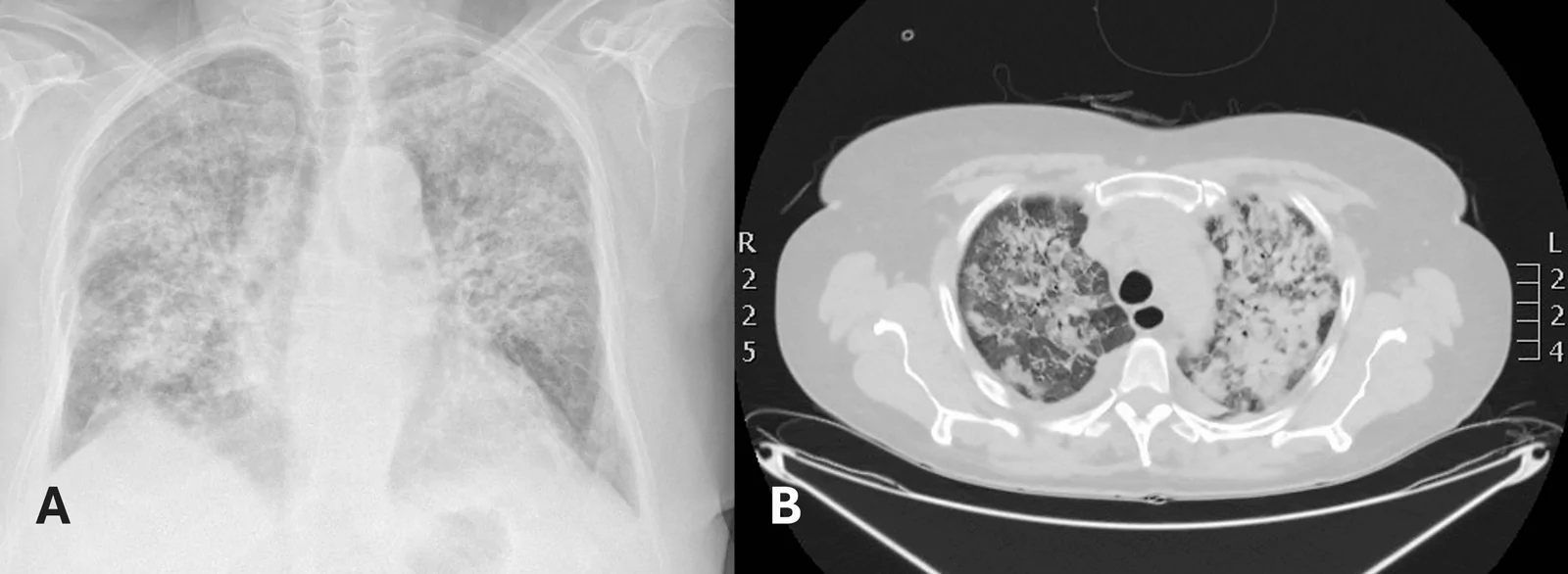
Chest imaging findings (A) Chest X-ray showing bilateral pulmonary opacities. (B) Chest computed tomography demonstrating bilateral ground-glass opacities with areas of consolidation, consistent with diffuse alveolar hemorrhage.

Since the clinical, laboratory, and radiological findings were indicative of a systemic vasculitis, a three-day high-dose pulse of intravenous methylprednisolone was initiated. In the meantime, MPO-p-ANCA returned positive, further supporting the working diagnosis of EGPA. In the absence of clinical improvement, treatment was escalated with the addition of rituximab (1000 mg). Given the ongoing alveolar hemorrhage, anticoagulation was switched from rivaroxaban to low-molecular-weight heparin, which was subsequently discontinued.

In the following days, hemoptysis increased, and hemoglobin levels progressively declined, requiring red blood cell transfusions. Five days after the first rituximab infusion (the second infusion was scheduled after two weeks), endotracheal intubation and transfer to the Intensive Care Unit (ICU) were mandatory due to rapid deterioration and severe respiratory distress. Her clinical status was critical, requiring deep sedation due to patient-ventilator asynchrony and demonstrating significantly reduced lung compliance. Treatment was further escalated with therapeutic plasma exchange (TPE) and intravenous cyclophosphamide pulses (500 mg/m², every three weeks). Eosinophilia resolved shortly after treatment initiation, but resolution of the alveolar hemorrhage was profoundly delayed. Owing to prolonged weaning, she was subjected to tracheostomy after 15 days of mechanical ventilation. She remained hospitalized in the ICU for a total of 35 days, followed by a period of rehabilitation in the ward.

## Discussion

Our patient was assessed in the Emergency Department, where clinical, radiological, and laboratory findings were suggestive of systemic vasculitis. The positive MPO-p-ANCA further supported the diagnosis, while the negative hematological work-up helped exclude an important alternative cause of eosinophilia. Moreover, BAL cell count revealed marked eosinophilia, and sequential BAL aliquots confirmed the suspected alveolar hemorrhage. In the “2022 American College of Rheumatology/European Alliance of Associations for Rheumatology Classification Criteria for Eosinophilic Granulomatosis With Polyangiitis” [7], a cumulative score ≥6 yielded a 99% specificity rate for the classification of EGPA in a validation data set consisting of various forms of vasculitis. In clinical practice, these criteria are applied for classification purposes once a small- or medium-vessel vasculitis has been confirmed and after mimicking conditions have been excluded. In our case, eosinophilia (5 points) and medical history of asthma (3 points) yielded a cumulative score of 8, exceeding the threshold for EGPA classification. Despite the absence of histopathological confirmation, the overall presentation was highly suggestive of EGPA.

EGPA progresses in three successive stages, which may overlap: the initial prodromal stage, characterized by asthma and rhinosinusitis; followed by an eosinophilic stage, in which end-organ infiltrates may appear; ending with the vasculitic stage, where small- and medium-vessel involvement becomes clinically visible [1,3]. Our patient had a known history of asthma, eosinophilia, and documented sinus mucosal thickening before initiating omalizumab for the treatment of urticaria. This sequence indicates that the prodromal and eosinophilic phases of the disease preceded exposure to omalizumab. If EGPA was already present, alveolar hemorrhage could be the vasculitic manifestation of an already established disease, which led the patient to the emergency department. The temporal association with omalizumab is therefore noted but cannot be interpreted as causal. It is further possible that the urticarial lesions for which omalizumab was prescribed represented a cutaneous manifestation of EGPA that was misdiagnosed, although, in the absence of a skin biopsy, this cannot be confirmed. 

Several case reports have described the development of EGPA in patients under treatment with omalizumab, but a direct pathogenetic link has not been proven [8]. Steroid weaning is a possible mechanism of unmasking a previously controlled EGPA, but this is not always the case [9]. Nonetheless, there are clinical data supporting the use of omalizumab in patients already diagnosed with EGPA for better asthma control, while advising caution during corticosteroid tapering [10,11]. Our patient was not receiving systemic corticosteroid treatment before the initiation of omalizumab and did not reduce the dosage of inhaled corticosteroids.

At the time of initial treatment, the disease was considered to be non-life-threatening, and rituximab was therefore selected for its favorable safety profile. However, following subsequent clinical deterioration, escalation of immunosuppressive therapy was deemed necessary, and cyclophosphamide was added to the treatment, as per guidelines for life-threatening disease [12]. The same reasoning informed our decision to add therapeutic plasma exchange, where the indication rested on alveolar hemorrhage with hypoxemia rather than on renal function. Guidance in this setting is not uniform. Although the results of the PEXIVAS trial did not demonstrate a benefit on the composite of death or end-stage kidney disease [13], and the European Alliance of Associations for Rheumatology (EULAR) guidelines recommend against the addition of plasma exchange in alveolar hemorrhage [12], current Kidney Disease: Improving Global Outcomes (KDIGO) practice points retain alveolar hemorrhage with hypoxemia as a setting where plasma exchange may be considered [14]. In our patient, plasma exchange was therefore added as an individualized decision in the setting of refractory hypoxemic respiratory failure.

## Conclusions

This case illustrates the diagnostic complexity of EGPA in a patient receiving omalizumab for chronic urticaria. Eosinophilia, late-onset asthma, and rhinosinusitis were present before the first administration of the antibody. Corticosteroid tapering after omalizumab initiation has been proposed as a possible mechanism of unmasking a pre-existing disease, but this does not apply to our patient, who did not receive systemic corticosteroids at any point and therefore did not undergo systemic corticosteroid tapering. Taken together, these observations suggest that EGPA was most likely present but unrecognized when omalizumab was started, although a contributory drug effect cannot be excluded.

Two points follow for clinical practice. First, unexplained persistent eosinophilia should trigger a thorough clinical and laboratory investigation, as it could be the first manifestation of a systemic disorder. Second, our patient's course demonstrates that EGPA may progress within days or weeks to organ- and life-threatening disease if unrecognized. Progressive decline required escalation to cyclophosphamide, with therapeutic plasma exchange added as an individualized adjunctive measure. Early recognition remains the only reliable means of avoiding this outcome. 
